# An Innovative Method for the Conservative Treatment of Idiopathic Scoliosis Using the GraviSpine Device According to the Concept of Spinal Reflex Balance

**DOI:** 10.3390/jcm13144044

**Published:** 2024-07-10

**Authors:** Marek Kluszczyński, Katarzyna Zaborowska-Sapeta, Ireneusz Kowalski, Ilona Sylwia Karpiel

**Affiliations:** 1 Department of Health Sciences, Jan Dlugosz University, 4/8 Waszyngtona, 42-200 Częstochowa, Poland; 2 Department of Rehabilitation and Orthopedics, School of Medicine, University of Warmia and Mazury in Olsztyn, 10-082 Olsztyn, Poland; katarzyna.zaborowska@uwm.edu.pl (K.Z.-S.); rehab@uwm.edu.pl (I.K.); 3 Regional Specialized Children’s Hospital in Olsztyn, 10-561 Olsztyn, Poland; 4 Łukasiewicz Research Network—Krakow Institute of Technology, The Centre for Biomedical Engineering, Zakopiańska Str. 73, 30-418 Kraków, Poland

**Keywords:** scoliosis, spinal deformity, body posture, physiotherapeutic scoliosis-specific exercises, PSSE, conservative scoliosis treatment

## Abstract

**Objectives:** Conservative treatment of idiopathic scoliosis is more effective as a result of early diagnosis in conjunction with the use of specific physiotherapy and bracing techniques. Our aim was to investigate the effectiveness of specific physiotherapy developed according to the concept of spinal reflex balance using the GraviSpine device. This study is a retrospective analysis of prospectively collected data. **Methods:** A total of 199 patients aged 6–17 years, with a mean age of 11.26 ± 3.35 years, including 168 girls (84.4%) and 31 boys (15.6%), out of a total of 830 patients treated for IS at the Scoliosis Treatment Center in 2014–2019 were included in the assessment, which was conducted according to the inclusion and exclusion criteria. The study group was divided into three age groups. Group A subjects were 6–9 years old; group B, 10–12 years old; and group C, 13–17 years old. The mean follow-up time was 28.71 ± 10.98 months. Treatment outcomes were compared, based on changes in the Cobb angle and the angle of trunk rotation before and after treatment, both within the groups and with respect to sex and curvature location, using the Wilcoxon signed-rank test. Cobb angle changes in patients were classified as improvements, stabilizations, or deteriorations according to the SOSORT criteria. **Results:** A majority of patients improved or stabilized after treatment, with 67%, 71%, and 90% of subjects in groups A, B, and C, respectively, achieving these results. In group C (the oldest children), a statistically significant reduction of −1.84° ± 6.88° (6.31%) in the mean Cobb angle was achieved after treatment. With regard to sex, improvements and stabilizations accounted for 81% of cases in girls and 61% in boys, respectively. With respect to location, statistically significant reductions in the Cobb angle were noted for thoracic and thoracolumbar spines of −2.2° ± 7.54° (10.17%) *p* = 0.022 and −2.2° ± 6.58° (6.36%) *p* = 0.049, respectively. A significant reduction in the mean angle of trunk rotation was obtained in the group and in three curvature locations. **Conclusions:** Based on the presented research findings, the utilization of the GraviSpine device as an adjunct to specific PSSE (physiotherapeutic scoliosis-specific exercises) physiotherapy and bracing in the management of pediatric patients with mild to moderate scoliosis appears to enhance treatment efficacy.

## 1. Introduction

Idiopathic scoliosis (IS), as a three-dimensional spinal deformity, manifests in apparently healthy children and can develop depending on many factors during each rapid growth period [1,2,3]. It can have negative effects on physical and psychosocial health, such as pulmonary complications and pain syndromes, as well as on the mental health of adults [4,5]. Current research indicates that scoliosis can be defined as a subclinical disease of the nervous system manifesting itself in the musculoskeletal system [6]. This approach draws attention to the possibilities of using the functional plasticity of the nervous and musculoskeletal systems to therapeutic effect, improving postural control deficits in order to inhibit the development of IS [7,8,9]. Associates involved in the SRS and SOSORT international scientific societies have developed uniform principles of conservative treatment according to the following scheme: early diagnosis, physiotherapeutic scoliosis-specific exercises (PSSE), bracing and education, and, in the event of failure of conservative treatment, surgery [10]. As a result of the efforts of many authors, efficacy has been demonstrated in publications, with sufficient evidence being offered for both PSSE methods [11] and bracing [12,13,14], as well as for the use of both methods combined [10,15]. The development of PSSE and its consistent application as an approach over the last 20 years have resulted in a 50% decrease in the number of operations, resulting in an unchanged incidence of the disease [16]. The peer-reviewed scientific literature indicates that a Cobb angle of scoliosis between 10 and 25° may be stabilized or even reduced by using the exercise methodology according to PSSE Schroth, SEAS, Lyon, and BSPTS in IS, while the positive effect of treatment in curvatures with larger Cobb angles is attributed to the combined effect of PSSE and bracing [12,13,14].

The presented approach to the conservative treatment of idiopathic scoliosis using the GraviSpine device (PHU Technomex Sp. z o.o., Gliwice, Poland) as proposed by the primary author Marek Kluszczyński, emphasizes early diagnosis and the initiation of rehabilitation at the prescoliotic stage [17,18,19,20,21,22]. Kluszczyński highlights the necessity of restoring the disturbed resting tension balance of the deep spinal muscles (e.g., transversospinal muscles) at the onset of scoliosis development, identifying this imbalance as the pathomechanism underlying IS development [23].

**Figure 1:**
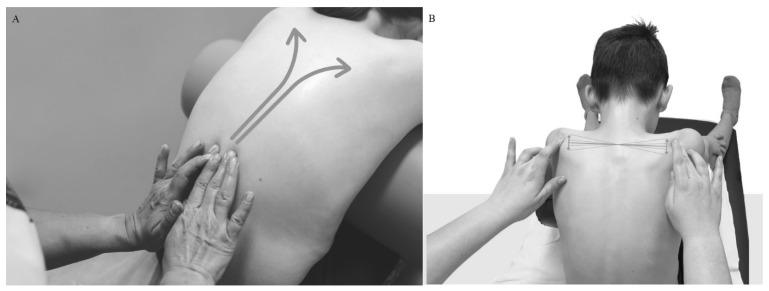
(**A**) Neurostimulation through slow, medium–strong pressure at points on both sides of the spine in the paraspinal line to the side from the spinous processes, simultaneously ascending, synchronously on the same segment of the spine, starting from S1 to Th 3. (**B**) Finishing with a smooth transition of fingers at the level of Th 3, the scapula comb to its center. The movement ending the maneuver is equalizing the level of the shoulder blades with a balancing movement, paying attention to keeping the head in the axis—posture re-education.

The described method, tentatively named spinal reflex balance (SRB), integrates elements from recognized PSSE (physiotherapeutic-specific exercises for scoliosis) methods such as Dobomed, Schroth, SEAS, Lyon, and FITS. It also employs active–passive scoliosis correction utilizing the GraviSpine device and tactile neurostimulation of the paraspinal area involving specific back massage in both lying and sitting positions (Figure 1). Derotational breathing exercises performed on the GraviSpine device, combined with passive curvature correction using lateral supports, actively stretch the deep paraspinal muscles and connective tissue structures, aiding in the balance of afferent input from sensory proprioceptors. Additionally, neurostimulation through massage contributes to the restoration of postural balance.

The aim of the study was to assess the effectiveness of the SRB method using the GraviSpine device in the treatment of IS on different age groups, as well as in terms of sex and curvature location.

## 2. Materials and Methods

### 2.1. Study Design: Retrospective Analysis of Prospectively Collected Data

The study was conducted at the Troniny Medical Rehabilitation Center and was based on the data of patients treated from 2014 to 2019. The patient and the parent/guardian were informed by the doctor about the method and procedures used and gave their written consent to the treatment. Qualification for treatment was based on X-ray evaluation in accordance with SOSORT [10] criteria of Cobb’s ≥10° scoliosis combined with vertebral rotation. The study group was treated on an outpatient basis according to a uniform scheme of treatments that occurred once a week at the center and for 20 min a day at home. The program at the center lasted 90 min, with each session comprising of 20 min of individual exercises based on the SRB concept, 20 min of back massage (paraspinal neurostimulation) combined with individual work of a physiotherapist on the trunk fascia (Figure 1A,B), two 20 min sessions of autocorrective breathing exercises on GraviSpine (separated by massage), and 10 min of learning corrected posture during daily activities.

**Figure 2:**
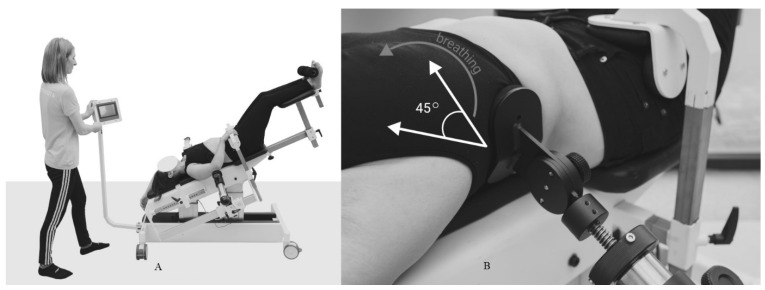
GraviSpine. (**A**) Antigravity spine stretching on GraviSpine and (**B**) asymmetric breathing exercise combined with passive curvature correction by pads.

For each child, the physiotherapist recommended two to three exercises to do at home in the period between meetings. In addition, the parent/guardian was instructed and obliged to massage the child’s back every day for 15 min, and, after the first program, physiotherapists checked the correctness of their performance. The patented GraviSpine device (Figure 2A,B) is a type of inversion lift for the lower limbs equipped with a system of movable side pads, indirectly correcting the curved section of the spine on three planes.

By acting directly on the child’s torso at the correct angle, the curvature of the spine is indirectly corrected. During the GraviSpine procedure, the child simultaneously performs derotational breathing exercises, which are supported by the action of pads. The device allows the child to lie on their back with their head down at an angle of approximately 20–30°. This position creates an antigravity environment to stretch the spine, which promotes relaxation of the facet joints and intervertebral space, facilitating three-plane correction of IS.

Each child was treated according to the same general scheme, which does not mean that each had the same exercises. The selection of exercises resulted primarily from the IS type but also from possible orthopedic deficits (muscle contractures, valgus knees, or tarsus) or sensorimotor deficits (disorders of the sense of posture, balance, and coordination).

Among all the children studied, 21% of children also underwent treatment with the Cheneau brace, as recommended by SOSORT [10] criteria (Cobb angle of the greatest curvature of scoliosis ≥ 20° and Risser grade 0–3). Despite the recommendations to wear the brace 23 h a day, the actual daily time worn was from 6 to 12 h, according to parental feedback. The period of bracing ranged from 6 to 38 months. Every three months, the doctor checked the progress of treatment by assessing the angle of trunk rotation (ATR) with a Bunnell scoliometer, the size of the anteroposterior curvature angles of the spine with the Saunders inclinometer, the functional length of the lower limbs, and the correctness of the massage performed by the parents. The patient’s height was also measured. In children treated with a brace, orthopedic technicians were also consulted by the doctor, in the presence of parents and children. An X-ray assessment of the spine was performed by a doctor once a year. The treatment was administered by a team of two doctors, eight physiotherapists, and two orthopedic technicians in close conjunction.

### 2.2. Participants

The inclusion criteria were newly diagnosed IS patients meeting the SOSORT criteria; participation in weekly visits with a minimum of two treatment cycles of 10 meetings; Risser grade 0–4; and at least one year of follow-up examinations.

The exclusion criteria were the occurrence of any secondary scoliosis (congenital, neurological, metabolic, post-traumatic, etc.), mental retardation, cardiovascular and respiratory diseases, and previous treatment of IS with other methods.

The study group that met the criteria consisted of 199 children, from a total of 830 treated at the center, in the age range 6–17 years, with an average age of 11.26 ± 3.35 years. There were 168 girls with an average age of 11.32 ± 3.2 years, and 31 boys with a mean age of 10.94 ± 3.05 years. Patients were divided into three age groups: group A, 6–9 years old, *n* = 57; group B, 10–12 years, *n* = 66; and group C, 13–17 years, *n* = 76, according to the divisions adopted in the center and in the literature [19].

### 2.3. Data Analysis

Cobb angle values were analyzed on initial and final X-rays. Radiographs were measured twice each time using standard radiographic computer software (RSR2LITE, 2-2.0.5.4) by a single medical specialist who was blinded to the subject data, and the test results were the average of both measurements. The percentage of improvement in the curve (Cobb angle decrease > 5°), stability (Cobb angle change ± 5°), and progress (Cobb angle increase > 5°) were compared [10]. The ATR value was analyzed on the basis of the first and final measurement results for the whole group, relative to the location of curvature. A nonparametric Wilcoxon signed-rank test was used to demonstrate differences between Cobb angle and ATR scores before and after treatment (dependent variables).

## 3. Results

The mean follow-up time was 28.71 ± 10.98 months. The size of the Cobb angle in the pretreatment group ranged from 10° to 46°, averaging 22.42° ± 12.01°. The ATR range was between 3 and 31°, with a mean angle of 5.28° (2.12°). The mean values of the Risser test for groups A, B, and C were 0, 1.38 ± 1.4, and 2.33 ± 1.1 degrees, respectively. The largest group included adolescents aged 13–17 (*n* = 76) (group C). Double-arch scoliosis predominated, with curvatures located in the thoracic and lumbar sections, exhibiting right-sided orientation in the thoracic section and left-sided orientation in the lumbar section (Table 1).

In this group, the mean reduction in Cobb angle after treatment was statistically significant, amounting to −1.84° ± 6.88° (−6.31%, *p* = 0.002). In group B, the mean Cobb angle after treatment also decreased, but not significantly (by −0.46° ± 8.95° (−8.28%)). In group A, the mean Cobb angle increased after treatment by a non-significant 0.69° ± 10.81° (15.57%) (Table 2).

The percentage of positive treatment effects increased with age as the most common improvement rate (25% of patients) and stabilization rate (65%) were found in group C, with worsening in only 10% of patients. Improvement and stabilization occurred slightly less often in group B, while the least improvement and stabilization rates were found in group A (the youngest group), at 23% and 44%, respectively (Table 3).

The mean Cobb angle decreased more in boys, by −2.6° ± 9.78° (−16.56%), while improvement and stabilization rates were greater in girls, at 23% and 57%, respectively (Table 4).

Depending on the location of the curvature, significant differences in Cobb angle changes were observed. In the thoracic spine, significant reductions of −2.2° ± 7.54° (10.17%) were reported in group C: improvement and stabilization were observed in 23% and 67% of patients, respectively, with worsening observed in only 10% of patients. Slightly worse thoracic spine results were recorded in group B, with improvement in 23% of patients, stabilization in 54%, and deterioration in 23%. In the youngest group (group A), improvements were observed in 22% of the patients, and either stabilization or progression was observed in 39% (Table 5).

In the thoracolumbar spine, there was also a significant reduction in the Cobb angle in group B, in which stabilization was noted in 87% of patients and progression in 13%. In the thoracolumbar section in group B, the mean Cobb angle decreased by −1.62° ± 7.07° (−3.24%), but the change was not statistically significant; a 62% majority of patients were stabilized, while improvement and deterioration accounted for 15% and 23% of the group, respectively (Table 6).

In groups A and B post-treatment, in the lumbar section, there was a slight increase in the mean Cobb angle of 1.5° ± 12.84° (26.51%) and 1.21° ± 9.39° (18.96%), respectively; these differences were not statistically significant. The best results were achieved in group C, with improvement observed in 39% of patients and stabilization observed in 51% (Table 7).

In the ATR assessment, there was a statistically significant (*p* = 0.001) reduction in the mean ATR for the whole group, from a baseline ATR of 5.28° (2.12°) to 4.51° (2.61°), with the greatest reduction (*p* = 0.001) found in the thoracolumbar localization from ATR 5.24° (1.94°) to ATR 4.07° (1.95°) (Table 8).

None of the 830 children treated at the center, including the study group (*n* = 199), required a referral for IS surgery over the six-year follow-up period.

## 4. Discussion

In the study, doctors and physiotherapists were unaware that their work would be evaluated so they focused solely on the needs of patients by strictly implementing treatment protocols. The risk of progression of IS [20] in the study group was between 40 and 75% in groups A and B and between 30 and 65% in group C.

The greatest efficacy of the SRB method was noted in patients from group C, where the mean Cobb angle decreased statistically significantly by −1.83° ± 6.88° (−6.31%), *p* = 0.002; improvement and stabilization occurred in 25% and 65% of patients treated, respectively; and worsening occurred in only 10%. In group B, there was a reduction in the mean Cobb angle of −0.46° ± 8.95° (−8.28%), but the difference was not statistically significant; improvements and stabilizations accounted for a total of 71% of the group. In the group of the youngest children (group A), there was a slight increase in the mean Cobb angle of 0.69° ± 10.81° (15.57%), although improvement and stabilization were achieved in a total of 67% of children.

**Figure 3:**
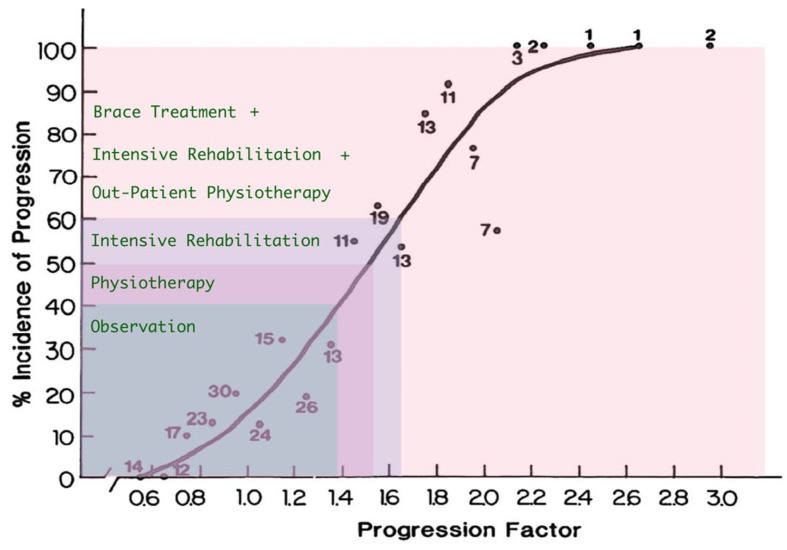
Estimation of the prognostic risk of IS progression (adapted from Lonstein and Carlson) [20,24], where “+” additional form of out-patient complementary treatment.

Due to the absence of a control group in the study, treatment outcomes were compared using the progression prediction method as delineated by Lonstein and Carlson [20] (Figure 3).

The progression estimation formula according to Lonstein and Carlson is given by:

(1) $$\frac{Cobb\;Angle-\left(3\times Risser\;sign\right)}{Chronological\;age}$$

The average progression factor was calculated for children from individual groups A, B, and C, yielding values of 2.59, 1.52, and 1.31, respectively. The calculations were performed as follows:

Group A: mean Cobb angle (19.2) − 3 × Risser sign (0), divided by the average age for the group (7.39 years) = 2.59; group B: mean Cobb angle (20.4) − 3 × Risser sign (1.38), divided by the average age for the group (10.67 years) = 1.52; and group C: mean Cobb angle (26.15) − 3 × Risser sign (2.33), divided by the average age for the group (14.67 years) = 1.31. Referring the values of progression factors for groups A, B, and C to the curve shown in Formula 1, the risk of scoliosis progression was 99%, 51%, and 35%, respectively.

These data illustrate that, particularly in children from groups A and B, there is a high likelihood of scoliosis progression if no treatment is initiated (observation only).

When comparing the data to the results of the effectiveness of other PSSE methods, it is noted that the majority (10) of RCT publications concern the Schroth method in combination with bracing treatment [21,22,23,25,26,27,28,29], among which the longest follow-up time (18 ± 6.2 months) and the group of similar age range (mean 12.3 ± 1.4 years) are characterized by the study of Kwan et al. [26]. The study found similar effectiveness of treatment, with improvement found in 17% of patients, stabilization in 62%, and progression in 21%. Slightly worse results were presented by Schreiber et al. [29], although the observation period was only 6 months. The study found a decrease in the Cobb angle of 0.4°, and a decrease in the Cobb angle range of the largest curve from −3.5° to −5° was obtained (*p* = 0.006). In the studies using PSSE by Schroth conducted by Kuru et al. [28], a more favorable Cobb angle reduction of −2.53° (*p* < 0.001) was obtained in the study group, while Otman et al. [30] achieved an even greater reduction of −6.85°, but it should be noted that in this study the observation periods were much shorter and the groups were smaller.

Park et al. [31] performed a meta-analysis of 15 publications on the effectiveness of the Schroth PSSE. The analysis showed that that the method was most effective in the treatment of minor scoliosis (10°–30° Cobb angle).

However, the comparison of the direct results of these studies is difficult due to the heterogeneity of the study data.

When comparing the results of our study to the results of long-term follow-up studies, i.e., the SEAS method, which, like Schroth, has a long tradition [32,33,34], it should be stated that the effectiveness of treatment in children, especially in group A, was similar because Negrini’s studies [35], showed an increase in the average Cobb angle of 1.7° ± 7.24°, which may be due to the fact that these children started therapy before puberty, after which it usually deteriorates, despite treatment [29].

The best results in the RTC study assessing the effectiveness of PSSE SEAS were presented by Monticone et al. [36], where the authors showed a Cobb angle reduction of −5.3° (*p* = 0.001) in the study group. Improvement was found in 69% of patients, stabilization in 39%, and worsening in only 8%. Shah et al. [23] demonstrated that even in shorter interventions (in this study, seven weeks), a positive effect due to IS treatment can be achieved via both the Schroth and SEAS methods. Comparing their effectiveness in 15 separate groups showed a reduction in the mean Cobb angle from 31.2° ± 5.2° to 27.4° ± 5.17° in the Schroth group and a reduction from 31.33° ± 5.26° to 29.4° ± 5.9° in the SEAS group. The difference between the methods was significant (*p* < 0.001). Comparison of the results of PSSE methods with a short observation period often does not allow objectification of the results by performing a control X-ray, and, although performed as an RCT, the conclusions should be approached with great caution.

Most authors unanimously point to the significantly better results of combined PSSE therapy and bracing in the PSSE SEAS [37], Schroth [23] combined with BSPTS [16,37,38], or FITS [39] combined with Dobomed. Negrini et al. [40] presented the statistical efficacy of treatment with PSSE and bracing combined, with control groups treated with only bracing or with bracing and general developmental exercises; however, the evidence that Negrini et al. used was of low quality. The study by Weinstein et al. [2] stands out as the highest scientifically rated publication in this field. The authors made a comparative analysis of the results of 116 patients in the bracing study group and 126 patients in the control (unbraced) group. In the analysis, which included both randomized and preferential cohorts, the success rate of bracing was 72%, compared with 48% after follow-up (odds ratio adjusted for propensity for treatment success, 1.93 and 95% confidence interval (CI), 1.08 to 3.46). In addition, there was also a significant positive correlation between the hours spent wearing the brace and the success rate of the treatment (*p* < 0.001).

In our study, 21% of children were treated with PSSE SRB and a Cheneau brace combined, and the others were treated only with PSSE SRB. The daily time spent wearing the brace was on average only 6–12 h, which, according to Weinstein [2], is only 40–70% effective.

A report on the use of a device supporting IS therapy in the literature was presented by Trzcińska et al. [41]. The study compared the results of three weeks of intensive treatment in the study group using the PSSE FITS in combination with the FED Dr. Sastre device, with the control group treated only with PSSE FITS (the device is manufactured by the company Sastre Instrumental Medico located in Valencia, Spain.). A statistically significant reduction in the ATR angle of the cervicothoracic spine and in relation to the sum of ATR in double-arch scoliosis was demonstrated. In our study, we obtained a statistically significant overall reduction in mean ATR, as well as in all three locations of curvature (Th, Th-L, and L).

The PSSE SRB model is distinguished from other methods by its early intervention (this is the reason why the study group includes children aged 6–9) [42], passive–active correction of scoliosis on the GraviSpine device [43], and the use of neurostimulation massage according to the concept of reflex balance. Inhibiting the development of scoliosis at an early stage by affecting the factors predisposing to its progression [44,45] allows us to achieve other important goals, such as improving neuromotor control, respiratory function, and stability of the joints of the limbs and spine, in order to improve the quality of life in adulthood [7,15,44,45,46].

The studies of Hawes and O’Brien [7], Burwell et al. [44], and Grivas et al. [45] form the basis of the above concept, as the authors indicated the need for curative modification of posture by improving the neurological control of its pattern at the beginning of IS formation, when the child is still in a prescoliotic state.

The strengths of the study include a long observation period at a medical center by experts with many years of experience, a fairly large evaluation group, and the use of innovative derotational breathing supported with three-plane passive curvature correction on the GraviSpine device.

The study has several limitations, including single-center design, lack of a control group, retrospective nature, and small sample size. Additionally, the validity of using neurostimulation massage is not supported by research and is based solely on the first author’s subjective experience.

In the future, we plan to conduct research with a control group treated with one of the recognized PSSE methods. Additionally, we aim to analyze changes in the Cobb angle during exercises on the GraviSpine and assess the durability of these effects. Furthermore, to validate the assumptions of the spinal reflex balance (SRB) method and the efficacy of neurostimulation performed during massage, we plan to conduct electromyographic tests and evaluate brain activity using functional magnetic resonance imaging (fMRI).

## 5. Conclusions

Based on the presented research results, the use of the GraviSpine device as an adjunct to PSSE and bracing in the treatment of children with mild and moderate scoliosis appears to improve treatment effectiveness.

## Figures and Tables

**Table 1:** Main patients’ characteristics.

Variable		Parameter		Value (%)
Age		N		199 (100%)
		Average (SD)		11.26 ± 3.35
		Median (Q1–Q3)		12 (10.25–15)
		Range		6–17
Gender		Girls		168 (84.4%)
		Boys		31 (15.6%)
Age group A (y)		6–9 (7.39 ± 1.13)		57 (28.6%)
Age group B (y)		10–12 (10.67 ± 0.81)		66 (33.2%)
Age group C (y)		13–17 (14.67 ± 1.25)		76 (38.2%)
Angle of Trunk Rotation (ATR) before treatment		N		199
		Average (SD)		5.28° (2.12°)
		Median (Q1–Q3)		6 (5–9)
		Range		3–16
Location of dominant curvature	Section	Left side	Right side	Total
	TH	8 (4%)	71 (35.7%)	79 (39.7%)
	Th-L	16 (8%)	23 (11.6%)	39 (19.6%)
	L	51 (25.6%)	30 (15.1%)	81 (40.7%)
Scoliosis type		Single arch		47 (23.9%)
		Double arch (more)		152 (76.1%)
X-ray Cobb angle before treatment (°)		Average (SD)		22.42° ± 12.01°
		Median (Q1–Q3)		21° (15°–30°)
		Range		10°–46°
Skeletal maturity according to the Risser test in groups		A		0
		B		1.38 ± 1,4
		C		2.33 ± 1.1
Duration of treatment and observation [months]		N		199
		Mean (SD)		28.71 ± 10.98
		Median (Q1–Q3)		18 (9–40)
		Range		1–67

**Table 2:** Patient Characteristics—total.

Group N = 199	(N)	Gender F/M	Age/* mAge [y]	** mRg	*** mCobb		**** Test	***** ACD(%)
					Before T	After T	*p*-Value	
A	57	47/10	6–9 7.39 ± 1.13	0	19.02 ± 9.17	19.71 ± 11.36	0.636	0.69 ± 10.81 (15.57)
B	66	55/11	10–12 10.67 ± 0.81	1.38 ± 1.4	20.42 ± 13.17	19.96 ± 12.28	0.654	0.46 ± 8.95 (8.28)
C	76	66/10	13–17 14.67 ± 1.25	2.33 ± 1.1	26.15 ± 10.6	24.32 ± 11.54	0.002	1.83 ± 6.88 (6.31)

* mAge [y]—mean age ± SD [years]; ** mRg—mean Risser grade ± SD°; *** mCobb—mean Cobb angle ± SD°; **** Test—Wilcoxon signed-rank test; and ***** ACD%—angle correction in degrees ± SD° (percentages %).

**Table 3:** Numbers and Percentage Values of IS Improvement, Stabilization, and Progression—total.

Group—(N)/% 199	Improvement		Stabilization		Progression	
	n	%	n	%	n	%
A—57/28.6	13	23	25	44	19	33
B—66/33.2	12	18	35	53	19	29
C—76/38.2	19	25	49	65	8	10

**Table 4:** Numbers and Percentages of Improvement, Stabilization, and Progress of IS —in relation to gender.

Gender	n/%	* mCobb b.t. ± SD	Mean Age ± SD [y]	Follow-up ± SD	** mCobb ch. ± SD%	Improvement		Stabilization		Progression	
						n	%	n	%	N	%
Male	31/15.6	23.17 ± 12.81	10.94 ± 3.05	20.77 ± 10.98	−2.61 ± 9.78 (−16.56)	6	19	13	42	12	39
Female	168/84.4	22.03 ± 11.33	11.32 ± 3.2	25.78 ± 15.45	1.26 ± 8.57 (−2.63)	38	23	96	57	34	20

* mCobb b.t. ± SD—main Cobb before treatment ± SD and ** mCobb ch. ± SD %—main curve change ± SD (percentages %).

**Table 5:** Patient characteristics with scoliosis in thoracic section—Th.

Group	(N)	Gender F/M	* mCobb			*** Test	Improv. n/%	Stab. n/%	Prog. n/%
			Before t	After t	** ACD*%*	*p*-Value			
A	23	19/4	20.78 ± 9.73	21.96 ± 12.2	−1.17 ± 11.42 (−15.8)	0.602	5/22	9/39	9/39
B	26	22/4	22.04 ± 15.91	20.42 ± 14.26	1.62 ± 9.36 (0.29)	0.345	6/23	14/54	6/23
C	30	26/4	27.47 ± 10.48	25.27 ± 12.99	2.2 ± 7.54 (10.17)	0.022	7/23	20/67	3/10

* mCobb—mean Cobb angle ± SD°; ** ACD%—angle correction in degrees ± SD° (percentages %); and *** test—Wilcoxon signed-rank test.

**Table 6:** Patient characteristics with scoliosis in thoracolumbar section—Th-L.

Group	(n)	Gender F/M	* mCobb			*** Test	Improv. n/%	Stab. n/%	Prog. n/%
			Before t	After t	** ACD%	*p*-Value			
A	14	11/3	15.36 ± 5.54	14.04 ± 5.74	1.31 ± 6.01 (0.44)	0.395	3/22	9/64	2/14
B	13	11/2	20 ± 7.57	18.38 ± 4.61	1.62 ± 7.07 (−3.24)	0.421	2/15	8/62	3/23
C	15	13/2	24.4 ± 9.46	22.2 ± 10.21	2.2 ± 6.58 (6.36)	0.049	0	13/87	2/13

* mCobb—mean Cobb angle ± SD; ** ACD%—angle correction in degrees ±SD (Percentages %); and *** Test—Wilcoxon signed-rank test.

**Table 7:** Patient characteristics with scoliosis in lumbar section—L.

Group	(N)	Gender F/M	* mCobb			*** Test	Improv. n/%	Stab. n/%	Prog. n/%
			Before t	After t	** ACD%	*p*-Value			
A	20	17/3	19.55 ± 10.17	21.1 ± 12.39	−1.55 ± 12.84 (−26.51)	0.575	5/25	7/35	8/40
B	27	22/5	19.07 ± 12.61	20.28 ± 13.05	−1.21 ± 9.39 (−18.96)	0.431	4/15	13/48	10/37
C	31	27/4	25.72 ± 11.38	24.42 ± 10.87	1.3 ± 6.53 (2.55)	0.256	12/39	16/51	3/10

* mCobb—mean Cobb angle ± SD; ** ACD%—angle correction in degrees ±SD (Percentages %); and *** Test—Wilcoxon signed-rank test.

**Table 8:** Changes in ATR after treatment grouped by scoliosis localization.

Section of the Spine/N	Parameter	Before t	After t	Test Wilcoxon/*p*-Value
Total/N(199)	Average (SD)	5.28 (2.12)	4.51 (2.61)	<0.001
	Median (IQR)	5 (4–6)	4 (3–5)	
	Range	3–31	3–31	
Thoracic Th/ N (79)	Average (SD)	5.4 (2.78)	4.83 (2.93)	<0.001
	Median (IQR)	5 (4–6)	4 (3–5)	
	Range	3–25	3–31	
Thoraco-lumbar- Th-L/N (42)	Average (SD)	5.24 (1.94)	4.07 (1.95)	<0.001
	Median (IQR)	5 (4–6)	4 (3–5)	
	Range	3–16	0–14	
Lumbar L/N (78)	Average (SD)	5.11 (2.87)	4.9 (2.41)	<0.001
	Median (IQR)	5 (4–6)	5 (4–6)	
	Range	3–31	3–25	

## Data Availability

The raw data supporting the conclusions of this article will be made available by the authors on request.
